# Decellularized Wharton’s Jelly and Amniotic Membrane Demonstrate Potential Therapeutic Implants in Tracheal Defects in Rabbits

**DOI:** 10.3390/life14060782

**Published:** 2024-06-20

**Authors:** Aloysio Enck Neto, Katia Martins Foltz, Thiago Fuchs, Luize Kremer Gamba, Marcos Antonio Denk, Paulo Cesar Lock Silveira, Thatyanne Gradowski do Nascimento, Alice Machado Clemencia, Julio César Francisco, Lucia de Noronha, Luiz César Guarita-Souza

**Affiliations:** 1 Graduate Program in Health Sciences, School of Medicine, Pontifícia Universidade Católica do Paraná (PUCPR), Curitiba 80215-901, PR, Brazil; katia_foltz@hotmail.com (K.M.F.); luizekremer@hotmail.com (L.K.G.); thaty.gfcn@gmail.com (T.G.d.N.); julio.apfr@gmail.com (J.C.F.); lnno.noronha@gmail.com (L.d.N.); guaritasouzalc@hotmail.com (L.C.G.-S.); 2 Veterinary Medicine Undergraduated Program, University of Contestado (UNC), Mafra 89300-000, SC, Brazil; thiago.fuchs@professor.unc.br; 3 Biomedicine Undergraduate Program, School of Medicine and Life Sciences, Pontifícia Universidade Católica do Paraná, Curitiba 80215-901, PR, Brazil; slidesdenk@gmail.com; 4 Program of Postgraduate in Science of Health, Laboratory of Experimental Physiopathology, Universidade do Extremo Sul Catarinense (UNESC), Criciúma 88806-000, SC, Brazil; psilveira@unesc.net (P.C.L.S.); alicemachadoclemencia@hotmail.com (A.M.C.)

**Keywords:** biomaterial, tissue engineering, scaffold

## Abstract

Background: Tracheal grafts have been investigated for over a century, aiming to replace various lesions. However, tracheal reconstruction surgery remains a challenge, primarily due to anatomical considerations, intraoperative airway management, the technical complexity of reconstruction, and the potential postoperative morbidity and mortality. Due to research development, the amniotic membrane (AM) and Wharton’s Jelly (WJ) arise as alternatives within the new set of therapeutic alternatives. These structures hold significant therapeutic potential for tracheal defects. This study analyzed the capacity of tracheal tissue regeneration after 60 days of decellularized WJ and AM implantation in rabbits submitted to conventional tracheostomy. Methods: An in vivo experimental study was carried out using thirty rabbits separated into three groups (Control, AM, and WJ) (n = 10). The analyses were performed 60 days after surgery through immunohistochemistry. Results: Different immunomarkers related to scar regeneration, such as aggrecan, TGF-β1, and α-SMA, were analyzed. However, they highlighted no significant difference between the groups. Collagen type I, III, and Aggrecan also showed no significant difference between the groups. Conclusions: Both scaffolds appeared to be excellent frameworks for tissue engineering, presenting biocompatibility and a desirable microenvironment for cell survival; however, they did not display histopathological benefits in trachea tissue regeneration.

## 1. Introduction

Clinicians and engineers have investigated tracheal grafts for over 100 years to replace different lesions [1]. Tracheal reconstruction surgery is still a challenge due to anatomical considerations, intraoperative airway management, the technical complexity of reconstruction, and the potential postoperative morbidity and mortality [2]. Among the central pathologies observed, prolonged intubation is the leading cause of laryngotracheal stenosis, followed by neoplasms [3,4]. The treatment may depend on the localization and extent of the tracheal lesions [2].

Available prostheses often suffer from drawbacks like inflammation, inadequate integration with the host tissue, and extrusion. Moreover, they fail to emulate the properties of natural tissue [5]. In this way, tissue engineering has emerged as a promising technique for reconstructing long-segment tracheal injuries and restoring the trachea’s structure and function [6]. To this end, researchers are exploring a decellularization method that maintains the trachea’s original structure and offers mechanical strength and biocompatibility [7,8].

The ideal substitute for tracheal reconstruction needs to present good biocompatibility and tolerability, no local or systemic toxic effect, and the ability to generate growth factors and cytokines favorable to organ and tissue regeneration [9].

Due to the development of research in regenerative medicine, the amniotic membrane (AM) and Wharton’s Jelly (WJ) arise as alternatives within the new therapeutic alternatives. These structures hold significant therapeutic potential for tracheal defects as they can serve as a substrate for tissue regeneration, effectively reducing undesirable scarring effects [9,10].

The AM is a biocompatible tissue with mechanical properties that make it a potential framework due to its stability, flexibility, and permeability. It can support cell migration, adhesion, and tissue development [11,12]. WJ is a gelatinous substance of connective tissue and mesenchymal cells, abundant in substances such as hyaluronic acid and some sulfated glycosaminoglycans immobilized in an insoluble network of collagen fibers [13]. WJ is a promising candidate for tissue-engineered trachea as it shares similarities with cartilage tissue in composition and function [14].

In previous studies, the use of decellularized structures has yielded promising results. For instance, a 2021 study demonstrated that the combination of bone marrow stem cells and decellularized human amniotic membrane, when tested in a heart failure model, showed potential for regenerating cardiac function [15]. Additionally, another study focused on the amniotic membrane revealed that including an anti-inflammatory molecule supported tissue regeneration in the trachea, leading to the development of immature cartilage and collagen formation, thereby preventing the reduction of the tracheal lumen [16]. Furthermore, research on using Wharton’s jelly in tracheal regeneration found that implantation did not elicit adverse reactions such as rejection and extrusion [17].

The studies mentioned all focused on a decellularized biomaterial choice and shared the common limitation of not assessing a period longer than 35 days. In this way, in the current study, we analyzed the capacity of tracheal tissue regeneration after 60 days of decellularized Wharton’s Jelly and amniotic membrane implantation in rabbits submitted to conventional tracheostomy.

## 2. Materials and Methods

This was an experimental, interventional, and randomized study with 30 male New Zealand rabbits with an average weight of 3.00–3.500 kg. The entire process was registered and approved by an ethics committee (registration 18/20) and followed all the guidelines of the Declaration of Helsinki.

The animals were housed in 80 cm^3^ cages, with only one animal per cage; the cages were air-conditioned with continuously adjustable temperature and exhaust air, under light/dark cycles (12/12 h). They were fed Funny Bunny Blend rabbit food with an approximate intake of 40 g/day per animal and water ad libitum.

**Figure 1:**
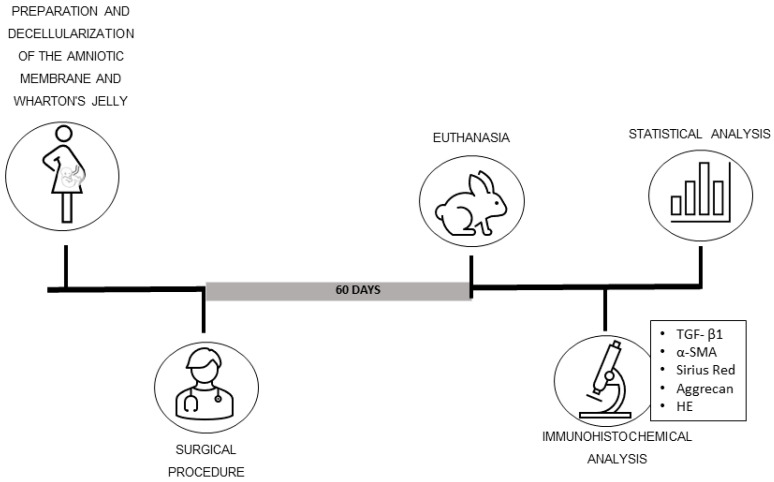
Experimental design.

The animals were randomly divided into three groups for analysis: Control Group (CTG) (n = 10): submitted to tracheostomy alone; Amniotic Membrane Group (AMG) (n = 10): submitted to tracheostomy and later implantation of AM at the tracheostomy site; Wharton’s Jelly Group (WJG) (n = 10): submitted to tracheostomy and later implantation of WJ at the tracheostomy site (Figure 1).

### 2.1. Preparation of the Umbilical Cord

Two pregnant women (n = 2) with gestational age between 36 and 40 weeks who had a vaginal delivery were selected. After the mothers signed the free and informed consent form approved by the Research Ethics Committee of Hospital Pequeno Príncipe under number 948-11, the maternal donors were tested to be serologically negative for HIV, hepatitis B, hepatitis C, and syphilis. The donated umbilical cords were processed within six to twelve hours after the delivery.

### 2.2. Decellularization of the Amniotic Membrane and Wharton’s Jelly

Decellularization was performed using an aseptic technique in a Class II BioSAFE (Veco^®^) biological. Thereafter, the sample was treated with chemical and mechanical processes using 0.01% SDS solution (sodium dodecyl sulfate) (Sigma Aldrich, Munich, Germany) and SD (sodium deoxycholate) (Sigma Aldrich, Munich, Germany) at 0.01% for 24 h at 37 °C in pH 7.2 saline phosphate buffer (PBS), with the aid of a stirrer mechanic (Shaking Table 109 M, Nova Ética Ltda, São Paulo, Brazil). Then, the sample was preserved in PBS (Gibco; Thermo Fisher Scientific, Inc., Waltham, MA, USA) at 4 °C, according to the methodology described by Blume et al., 2021 [15].

### 2.3. Surgical Procedure

All surgical procedures on the animals were conducted under the effect of anesthetics. The animals were fasted for 6 h on food and 2 h on water. The animals were placed in dorsal decubitus with manual physical restraint. Two-lead electrocardiography monitoring and a 100% oxygen supply were then installed. Induction of anesthesia was carried out with acepram at a dose of 0.75 mg/kg and ketamine at a dose of 35 mg/kg, both intramuscularly (IM). Five mg/kg Xylazine IM was used for muscle relaxation.

The surgery started with antisepsis of the cervical region with topical iodopolyvidone (PVPI), sterile drapes, local anesthesia with 2% lidocaine, without vasoconstrictor (dose of 1 mg/kg), waiting for a latency time of 5 min and respecting an effect time of 30 min. Median (longitudinal) cervicotomies were performed after dissecting the trachea, preserving its lateral portion, which is responsible for its vascularization.

At the time of surgery, tracheostomy was performed on the anterior portion of the trachea, resecting a rectangle (10 mm wide by 20 mm long) measured with a sterile ruler.

The membrane was washed in PBS containing antibiotics as well as antifungal (100 U/mL penicillin, 100 mg/mL streptomycin) and antimycotics (2.5 µg/mL amphotericin B) agents to reduce the microbial load. Then, immediately afterward, the animals in the AMG underwent implantation of a portion of amniotic membrane over the tracheal defect created, and the WJ group underwent implantation of a piece of Wharton’s Jelly over the tracheal defect created. A rectangular section of Wharton jelly, measuring 3 × 5 mm (15 mm^2^), was applied over the tracheostomy.

The suture was supported by four repair stitches at the ends of the rectangle using a 4.0 needle polypropylene thread. After the suture was completed, pre-tracheal muscles were approximated by a continuous suture using a number 2.0 needle cotton thread.

**Figure 2:**
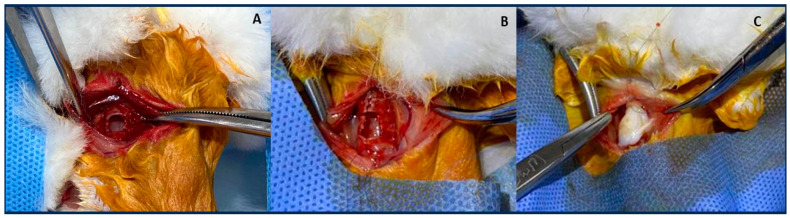
Surgical procedure. In (**A**) Control group: only tracheostomy; in (**B**) Amniotic Membrane Group: implantation of a portion of amniotic membrane over the tracheostomy; in (**C**) Wharton’s Jelly group: implantation of a portion of Wharton’s Jelly over the tracheostomy.

The CTG animals only underwent tracheostomy, and the conduit was kept open for healing by the second intention. Subsequently, pre-tracheal muscles were approximated, and the subcutaneous cellular tissue and skin were closed (Figure 2).

Postoperative analgesia was carried out throughout the 60-day recovery and observation period, using carprofen at 4 mg/kg/day subcutaneously (SC) once a day for five days, as well as morphine at a dose of 2 to 5 mg/kg SC every 4/4 h during the surgical procedure and in the postoperative period as required.

Other postoperative care included prophylactic antibiotic therapy, dressings, and routine assessment of the surgical wound. For antibiotic therapy, Cephalexin was given at a dose of 15 mg/kg SC every 12 h for seven days.

### 2.4. Euthanasia

All the animals were euthanized after an observation period of 60 days. General anesthesia was performed in the operating room in the vivarium under the care of veterinarian Dr. T.F. (CRMV 5549) using acepram, xylazine, and ketamine in the pre-determined doses, followed by an infusion of potassium chloride at a dose of 100 mg/kg intravenously (IV). Respiratory rate (RR) and heart rate (HR) were monitored until they were absent. The animals were removed by a company specializing in biological waste disposal (under the responsibility of GR Environmental Solutions).

### 2.5. Histopathology

For the histological study of the anatomopathological patterns of the animals, the lesion samples were placed in plastic containers and labeled; they contained 10% paraformaldehyde for 48 h and were sent to the experimental pathological anatomy laboratory at PUCPR to be processed using the paraffin embedding technique, were cut, and were stained.

The histological sections were stained with Sirius Red (Direct Red: Aldrich Chemical Company Inc., Milwaukee, WI, USA) to evaluate the deposition of collagen I (mature) and III (immature) and Hematoxylin and Eosin (HE) (Harris Hematoxylin: NewProv, Cod. PA203, Paraná, Brazil; Eosin: BIOTEC Reagentes Analíticos, Cod. 4371, Paraná, Brazil) for histological evaluation at the site of the lesion.

The slides were photographed at a magnification 400 (high power field or HPF) under polarized light. The markers were evaluated using Image-Pro Plus 4.5 software (Media Cybernetics, Rockville, MD, USA), where the polarized areas were identified (red for mature collagen I or green for immature collagen III). The collagen I (mature) and III (immature) analysis values were expressed as a percentage per HPF.

### 2.6. Immunohistochemical Analysis

The immunohistochemistry test was preceded by preparing multi-sample paraffin-embedded tissue blocks, TMA (Tissue Microarray). The pieces were ready on slides stained with different α-SMA, TGF-β1, and Aggrecan markers for the immunohistochemistry technique.

The immunohistochemistry technique begins with a protocol for incubating the primary antibodies in a humid chamber (at a temperature of between 2 and 8 °C, overnight) and using a secondary polymer (Mouse and Rabbit Specific HRP/DAB IHC Detection Kit-Micropolymer, Abcam, ab236466, Cambridge, UK) at room temperature. The technique was revealed by adding the 2, 3, diaminobenzidine complex + hydrogen peroxide substrate (Table 1). The reactivity of positive and negative controls confirmed the results. The slides were then scanned with a Zeiss Axioscan scanner (Jena, Germany) in high-power fields (HPFs; 20× magnification).

### 2.7. Statistical Analysis

Quantitative results were represented using the mean and standard deviation. Statistical analysis of variance was carried out using ANOVA, followed by the Kruskal–Wallis test to compare the groups. Values where * *p* < 0.05 were considered significant. The analyses were carried out using the Graph Pad Prism 9.0 program (San Diego, CA, USA).

## 3. Results

The animals used in this study were separated into three groups (Control, Amniotic Membrane, and Wharton’s Jelly), and all groups started this study with n = 10. However, during the analysis of the animal groups, there were losses due to infestation by Sarcoptic mange, where the animals were treated for 30 days with advokt (0.4 mL) and for behavioral stress.

**Figure 3:**
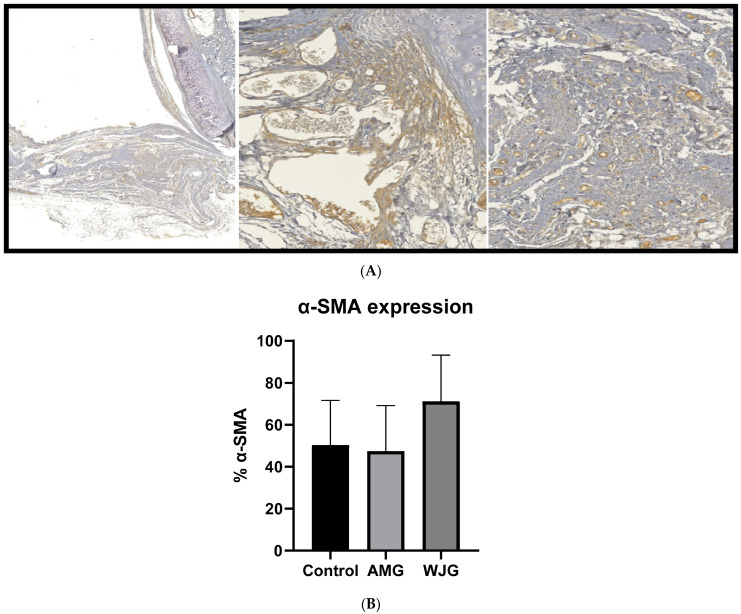
Expression of α-SMA marker in CTG, AMG, and WJG. Panel (**A**) displays the marker’s expression in tracheal tissue. In Panel (**B**), the results are presented with mean and standard deviations. ANOVA test was followed by the Kruskal–Wallis test.

Only the best tracheal segments from each group were chosen for the immunohistochemical analysis using HE staining. Slides with unreadable artifacts or cuts were excluded. In order to analyze the epithelial–mesenchymal transition and the arrangement of the extracellular matrix in healing wounds and scars, slides stained with α-SMA were used. Two images of each slide were taken, and a final percentage was generated based on these images. Statistical analysis was performed using the mean percentage for each group. The average percentage in the SMA CTG was 50.39% (SD ± 21.28); in the AMG, it was 47.43% (SD ± 21.79); and in the WJG, it was 71.17% (SD ± 22.03). These results indicate that the use of decellularized Wharton’s Jelly may lead to a higher percentage of an epithelial–mesenchymal transition and extracellular matrix arrangement, suggesting a more favorable environment for tissue regeneration. However, no significant difference was observed when comparing the groups, indicating that further research is needed to fully understand the implications of these findings (Figure 3).

**Figure 4:**
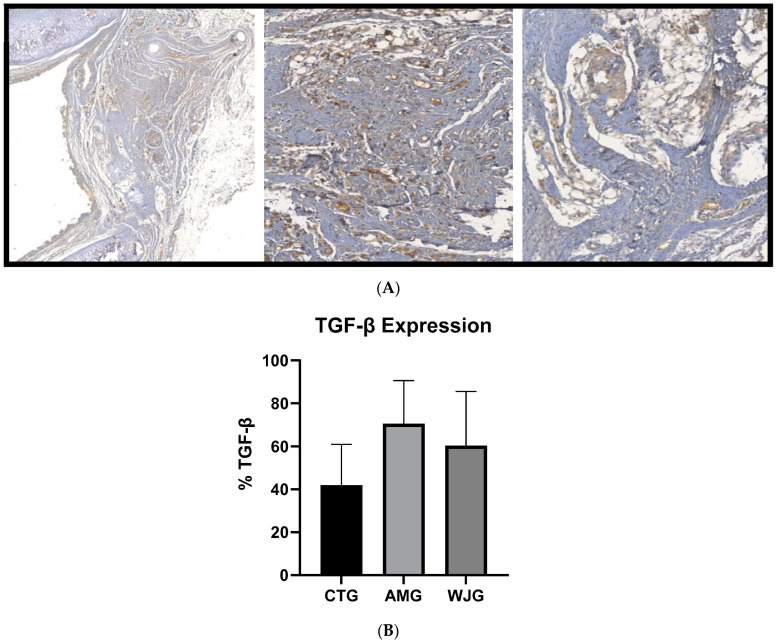
Analysis of TGF-β immunoexpression in the three groups analyzed (CTG, AMG, and WJG). Part (**A**) displays the marker’s expression in the tracheal tissue examined. Part (**B**) shows the results with mean and standard deviations. ANOVA test followed by Kruskal–Wallis test.

The same grouping was used to analyze the expression of TGF-β1, a multifunctional cytokine that plays a crucial role in tissue recovery after injury. It was found that for the TGF-β CTG, the average was 41.94% (SD ± 19.04); for AMG, the average was 70.5% (SD ± 20.16); and for WJG, the average was 60.41% (SD ± 25.12). However, the groups displayed no significant differences (Figure 4).

**Figure 5:**
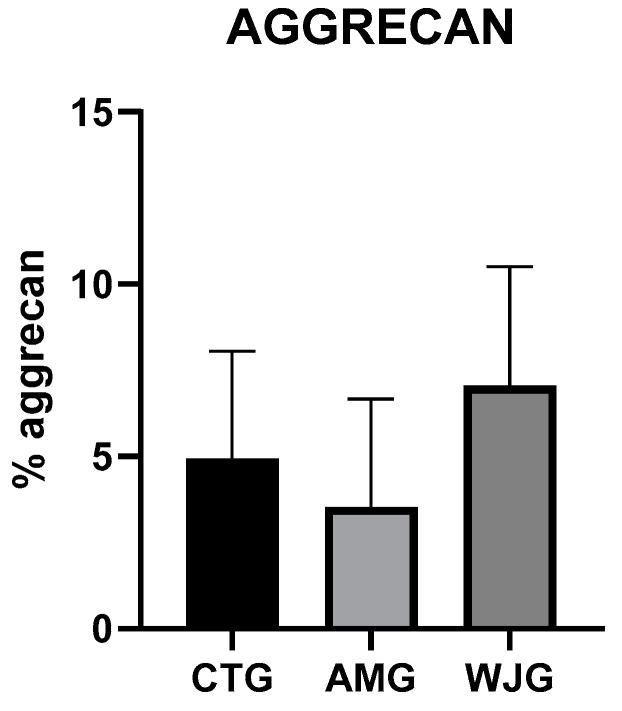
Immunoexpression of Aggrecan was analyzed in three groups: CTG, AMG, and WJG. Results are presented as mean and standard deviation. ANOVA test followed by Kruskal–Wallis test.

In the analysis of cartilaginous proliferation in the tracheal tissues using the Anti-Aggrecan antibody, the mean percentage for the Control group was 4.93% (SD ± 3.11); for the AMG, it was 3.52% (SD ± 3.14); and for the WJG, it was 7.052% (SD ± 3.44). No significant differences were observed between the groups (Figure 5).

This study checked the production of type I and III collagens. The results showed that for collagen I, the mean production percentage was 75.86% (SD ± 8.46) for the CTG, 66.87% (SD ± 12.48) for the AMG, and 74.86% (SD ± 6.83) for the WJG. However, there was no significant difference in the production of these collagens between the groups.

**Figure 6:**
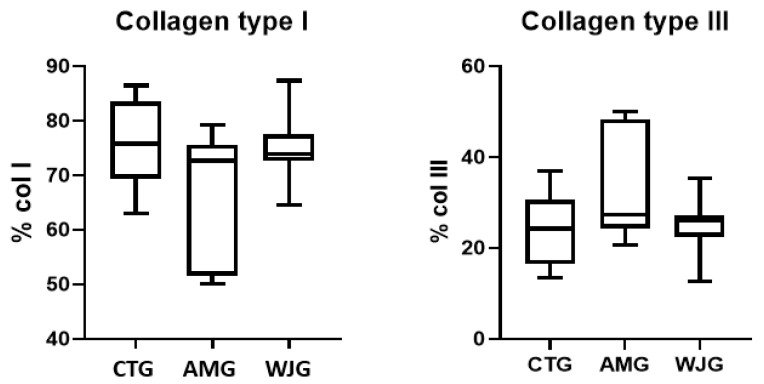
Analysis of collagens I and III in the three groups analyzed (CTG, AMG, and WJG). All the results were presented with mean and standard deviations. ANOVA test followed by Kruskal–Wallis test.

Similarly, for collagen III, the mean production percentage was 24.14% (SD ± 8.46) for the CTG, 33.13% (SD ± 12.48) for the AMG, and 25.14% (SD ± 6.83) for the WJG. Again, there was no significant difference in the production of collagen between the groups (Figure 6).

The perimeter and tracheal areas of the animals were also measured based on HE staining, which was utilized to evaluate measurements by analyzing the area and size of a defect. No statistical difference was observed between the groups in any of the variables (Table 2).

## 4. Discussion

Tracheal resection after an injury is considered a safe procedure. However, it still represents a significant challenge due to postoperative complications that can seriously affect patients’ quality of life [8]. In this context, researchers have increasingly investigated decellularized tissue-based biological structures as an alternative for restoring the structure and function of the trachea after surgical procedures [5]. Different immunomarkers related to scar regeneration, such as aggrecan, TGF-B1 and a-SMA, were analyzed to understand cell behavior in contact with the chosen structures. Both Wharton’s Jelly and the amniotic membrane have proven to be excellent frameworks for tissue engineering, presenting biocompatibility and a desirable microenvironment for the survival of different cells.

The healing process, whether caused accidentally or surgically, involves several phases of tissue repair, such as coagulation, inflammation, proliferation, and remodeling. Dysregulation of one of these processes can lead to poor healing, such as excessive scarring [18]. Several processes are involved in the formation of these scars, such as abnormal cell proliferation, the transformation of fibroblasts in response to damage, the stimulation of cytokines such as TGF-β, and the regulation of genes such as α-SMA, which are involved in the extracellular matrix, as well as the production of collagen and fibronectin.

TGF-β is involved in several scar formation processes, such as promoting the synthesis of extracellular matrix (ECM) components, including collagen and fibroblast proliferation, as well as inducing fibroblast differentiation. It also participates in scar formation by regulating fibrosis and keratinocyte proliferation [19]. It is also considered a crucial mediator in tissue fibrosis and causes scar formation in tissues [20].

Another aspect to consider is the role of TGF-β1 in the differentiation and activation of myofibroblasts, which induce the expression of α-SMA. The highest expression of α-SMA was in the Wharton’s Jelly group, but it displayed the lowest expression of TGF-β1 compared to the control group. However, when comparing the same biomarker, the values are very close, which could be associated with the connection between the two markers. Despite the MAG displaying a higher expression of TGF-β1, it did not show a higher expression of α-SMA.

The expression of α-SMA can be related to the wound’s contraction power and the development of excessive fibrotic reactions that manifest as a “bad scar” [20]. The advantage of both analyzed structures is their three-dimensional structure, which allows perfusion and vascularization, enabling cell repopulation to occur at the injury site, allowing the tissue to regenerate correctly [6].

The main chemical components of cartilage are water, collagen, and proteoglycans [21]. Although type II collagen is the main constituent of cartilage tissue, cartilage engineering approaches are mainly based on type I collagen devices [22].

In the analysis of aggrecan, the largest proteoglycan found in cartilage [22], it was possible to observe that the WJG or AMG were similar to the control after 60 days of analysis. Foltz et al., 2022, in a similar study, which analyzed the properties of Wharton’s Jelly for 30 days in rabbits after a tracheal injury, suggested that despite its various characteristics as a biomaterial, this structure did not present histopathological benefits for the regeneration of tracheal tissue. In this study, cartilage formation was evaluated using the same Aggrecan marker, where there was no significant difference between the control and the Wharton’s Jelly groups over 30 days [17]. In another study, Simeoni et al. (2021) evaluated a decellularized amniotic membrane as a framework for tracheal lesions using mesenchymal stem cells, finding cartilaginous tissue through the formation of immature collagen in the regions of the tracheal defects after 60 days [23].

Type I and type III collagens participate directly in wound healing and tissue regeneration, with an increase in type III collagen synthesis during the initial phases and an increase in type I collagen synthesis during the final phases [24]. The remodeling process can occur from 3 weeks to 1 year after the injury [25]. During the proliferative phase, also known as the tissue regeneration phase, extracellular matrix replacement, angiogenesis, and epithelization occur, associating factors such as transforming growth factor-β (TGF-β) and components such as collagen III [26]. In the results obtained, the rates of type I collagen found were similar between the groups. As for type III collagen, there was a slight increase in the MA group, which may corroborate the suggestion that using biomaterials leads to a delayed healing process in the observed tissue. These results corroborate another study that used an amniotic membrane, which showed that, after 35 days, there was no difference between the group that used only the membrane and the group that used the membrane as well as an anti-inflammatory molecule [16].

Some of the challenges we faced included keeping the animals alive for a specific duration and the occasional loss of an animal. Additionally, we could only analyze the final 60 days of this study and we could not continually monitor the rates of the biomarkers under investigation. It would have been helpful to have markers that could be tracked throughout this study.

Different strategies can be used for tracheal regeneration—insemination of cells on an implanted scaffold, implantation of tissue grown in vitro on a scaffold, or scaffold implantation without cells to support tissue regeneration in situ—to allow the new tissue to grow and for it to present biological properties compatible with the implanted site [8].

Understanding the roles of various molecules involved in extracellular matrix (ECM) remodeling in tissue injury and regeneration is pivotal in evaluating frameworks for tracheal regeneration. This study thoroughly examined the formation of cartilaginous tissue over 60 days. The extended duration of this study offers a more comprehensive understanding of the potential of these structures in the regeneration of tracheal tissue. This study evaluated the ability of aggrecan to form an adequate cartilaginous matrix and type I and III collagens to support the regeneration and repair of tracheal tissue by assessing their strength and flexibility.

Moreover, it identified tissue remodeling activity correlated with reducing scarring and stenosis through smooth muscle actin (α-SMA). This study culminated in a microenvironment analysis to evaluate chronic inflammation and promote tissue reconstruction with TGF-β1. These markers allowed for a robust understanding of implant behavior within the biological environment, thus substantiating their potential as a viable therapeutic modality for tracheal defect reconstructions.

## 5. Conclusions

Our research has shown promising results, with both frameworks demonstrating the potential to restore the mechanical characteristics of the trachea’s extracellular matrix. These biomaterials provided the required support and environment to regulate the healing process of any remaining defects, thus preventing stenosis and maintaining the desired tracheal diameter. However, although they created a favorable microenvironment, they did not show significant histopathological benefits compared to natural tracheal regeneration.

However, there is still much to learn about the trachea’s recovery process, pathophysiology, and the molecular mechanisms associated with recuperation post-injury, presenting exciting opportunities for further investigation.

## Figures and Tables

**Table 1:** Antibodies description.

Antibody	Type	Clone	Dilution	Label
Anti-α-SMA	Monoclonal/Rato	BSB-15	1:600 pH 9	BioSB
Anti-TGF-β1	Policlonal/Coelho	E11262	1:200	Spring
Anti-Aggrecan	Monoclonal/Rato	Ab3773 BC3	1:200	Abcam

**Table 2:** Characterization of the rabbit population.

	Control Group (n = 7)	Wharton Jelly Group (n = 7)	Amniotic Membrane Group (n = 5)	*p*
Tracheal perimeter (µm^2^)	15,389 ± 1961	15,562 ± 1390	15,257 ± 2210	>0.9
Tracheal area (µm^2^) *	15,523,727 ± 4,086,295	14,712,626 ± 2,408,509	15,436,725 ± 4,217,003	>0.9

* Mean and standard deviation.

## Data Availability

The data that support the findings of this study are available from the corresponding author upon reasonable request.
